# Quantitative Assessment of Extrinsic Tongue Muscle Stiffness in Obstructive Sleep Apnea Using Shear Wave Elastography

**DOI:** 10.3390/diagnostics16010087

**Published:** 2025-12-26

**Authors:** Hilal Er Ulubaba, Nurullah Dağ, Sevgi Demiröz Taşolar, Rukiye Çiftçi, Hilal Ermiş

**Affiliations:** 1 Department of Radiology, Inonu University, Malatya 44280, Turkey; nurullah.dag@inonu.edu.tr (N.D.); sevgi.tasolar@inonu.edu.tr (S.D.T.); 2 Department of Anatomy, Gaziantep Islam Scıence and Technology Unıversıty, Gaziantep 27260, Turkey; rukiyekelesciftci@hotmail.com; 3 Department of Chest Diseases, Inonu University, Malatya 44280, Turkey; hilal.ermis@inonu.edu.tr

**Keywords:** obstructive sleep apnea, genioglossus muscle, geniohyoid muscle, ultrasonography, shear wave elastography

## Abstract

**Background/Objectives:** This study aimed to quantitatively and noninvasively evaluate the changes in the Genioglossus (GG) and Geniohyoid (GH) muscles in patients with Obstructive Sleep Apnea (OSA) using ultrasonography (US) and shear wave elastography (SWE). **Methods:** This prospective study included 94 adults (18–73 years) who underwent polysomnography (27 normal; 67 OSA). GG and GH muscle thickness was measured with US, and stiffness with SWE. Participants were grouped as non-OSA (Group 0) and OSA (Group 1). OSA patients were further divided by apnea–hypopnea index (AHI) into mild, moderate, and severe (Groups 1–3), forming four groups including controls. **Results:** No significant differences were observed in genioglossus or geniohyoid muscle thickness between groups. Shear wave elastography revealed significantly higher stiffness values for both the genioglossus and geniohyoid muscles bilaterally in OSA patients compared with non-OSA individuals (approximately 2.7 m/s vs. 2.4–2.5 m/s, *p* < 0.01). Geniohyoid muscle stiffness on both sides increased progressively with OSA severity, with significantly higher values in severe compared with mild OSA (*p* < 0.05). In contrast, genioglossus stiffness did not differ significantly across OSA severity subgroups. **Conclusions**: In patients with OSA, GH and GG muscle thickness remains unchanged, but their stiffness measured by SWE increases. GH stiffness also rises with increasing disease severity. These results indicate that GG and GH muscle stiffness may serve as useful noninvasive markers for OSA.

## 1. Introduction

Obstructive Sleep Apnea (OSA) is a common disorder characterized by recurrent collapse of the upper airway (UA), leading to hypopneas and apneas during sleep [1]. OSA affects at least 3–7% of the general population; however, it has been reported that 80–90% of these cases remain undiagnosed [2]. OSA can cause frequent awakenings, leading to inappropriate sympathetic activation and sleep fragmentation. Over time, adult patients with OSA often experience intermittent hypoxemia during sleep. Therefore, untreated OSA is associated with long-term health consequences, including cardiovascular and cerebrovascular diseases [1], metabolic disorders [3], and neurocognitive impairment [4]. Moreover, untreated OSA has also been linked to occupational and motor vehicle accidents that may result in injury or death [5]. The etiology of OSA includes factors such as male sex, advanced age, obesity, pregnancy, menopause, fluid retention, retrognathia, disproportionate craniofacial structures, and other muscular malformations [6]. The most common sites of pharyngeal airway collapse in OSA are the soft palate, lateral pharyngeal wall, epiglottis, and most importantly, the tongue or tongue base. Malformations of the muscles within the tongue are among the major causes of airway collapse [7].

The genioglossus (GG) and geniohyoid (GH) muscles are among the primary muscles responsible for maintaining upper airway patency by controlling the position of the tongue and hyoid bone. The GG muscle prevents contact between the tongue and the posterior pharyngeal wall by advancing the tongue forward, while the GH muscle stabilizes the tongue base by pulling the hyoid bone anteriorly and superiorly. Coordinated activity and adequate muscle tone of these two muscles are critical for maintaining airway patency, particularly during sleep [8]. Changes in the biomechanical properties of these muscles, particularly reduced elasticity, may disrupt their coordinated function and dynamic response to negative inspiratory pressure, leading to upper airway collapse in patients with OSA. Although previous studies have primarily focused on the genioglossus muscle, the simultaneous assessment of the genioglossus and geniohyoid muscles represents a novel approach that provides a more comprehensive understanding of the muscle-based mechanisms responsible for upper airway collapse in OSA [9].

Most previous imaging and elastography studies conducted in OSA have focused on the entire tongue or a single muscle, most commonly the genioglossus muscle. However, the patency of the upper airway depends on the coordinated function of multiple extrinsic tongue muscles. In this context, the simultaneous assessment of the genioglossus and geniohyoid muscles may provide more comprehensive information regarding upper airway biomechanics and contribute to the understanding of the muscle-based mechanisms of OSA [9].

Conventional B-mode ultrasound enables the assessment of muscle morphology, including muscle thickness and structural appearance; however, it provides limited information about the biomechanical properties critical for muscle function. Shear wave elastography (SWE) is an advanced ultrasound-based imaging technique that enables quantitative, objective, and non-invasive assessment of tissue elasticity, thereby providing an indirect insight into muscle biomechanical behavior [9]. Unlike strain elastography, SWE provides absolute, quantitative values, does not require reference tissue, and is significantly less operator-dependent, thereby increasing its reproducibility and clinical applicability [10]. Data on the objective and quantitative evaluation of the elasticity characteristics of the GG and GH muscles in OSA patients are limited. SWE, as an advanced imaging method that can be used for this purpose, has the potential to provide direct information about the functional status of these muscles by measuring their stiffness [11].

Therefore, the assessment of the genioglossus (GG) and geniohyoid (GH) muscles using shear wave elastography (SWE) may provide clinically meaningful information about their functional status and contribution to upper airway stability in patients with obstructive sleep apnea. Although there are a limited number of studies in the current literature, most have focused solely on the genioglossus muscle. Consequently, the simultaneous assessment of both the GG and GH muscles using SWE fills an important gap in the literature and allows for a more comprehensive evaluation of the muscle-based mechanisms underlying upper airway collapse. This study aims to elucidate the muscle-related pathophysiology of OSA by combining ultrasound with shear wave elastography and to determine the potential role of this non-invasive imaging technique in diagnosis.

## 2. Materials and Methods

### 2.1. Study Design

This single-center, prospective cross-sectional study was approved by the Inonu University Scientific Research and Publication Ethics Committee, Health Sciences Scientific Research Ethics Committee (Approval No.: 2025/7819, 24 June 2025), and was conducted in accordance with the Declaration of Helsinki, with written informed consent obtained from all participants. Informed consent was obtained from all subjects involved in the study. Between June 2025 and November 2025, patients aged 18–73 years who underwent polysomnography at the Pulmonology Sleep Clinic, both with and without a diagnosis of obstructive sleep apnea, were included in the study. A total of 67 individuals with OSA (AHI > 5/h on polysomnography) and 27 individuals without OSA (AHI < 5/h) were enrolled. The non-OSA participants were individually matched to OSA patients based on age, sex, and body mass index (BMI); sex was matched exactly, while age was matched within ±5 years and BMI within ±2 kg/m^2^. The 67 OSA patients were further divided into three subgroups according to AHI: mild OSA (*n* = 19), moderate OSA (*n* = 22), and severe OSA (*n* = 26). Exclusion criteria for participants included neuromuscular disease, syndromic craniofacial abnormalities, history of neck burns or trauma, upper airway or neck surgery, and prior radiotherapy.

### 2.2. Clinical Evaluation

All participants underwent overnight polysomnography (PSG) in a dedicated sleep laboratory using standard diagnostic protocols in accordance with the American Academy of Sleep Medicine (AASM) guidelines. Sleep stages and respiratory events were evaluated by an experienced sleep physician.

All participants underwent full-night attended polysomnography (PSG) in a dedicated sleep laboratory using a 55-channel computerized PSG system (Alice 6, Respironics; Philips, IL, USA). The recorded parameters included electroencephalography (EEG), electrooculography (EOG), submental electromyography (EMG), tibialis anterior EMG, electrocardiography (ECG), oronasal airflow (measured using a nasal pressure transducer), thoracoabdominal respiratory movements (assessed using thoracic and abdominal belts), arterial oxygen saturation (measured by finger pulse oximetry), body position sensor, and snoring sensor.

PSG recordings were manually analyzed by an experienced physician certified in sleep medicine. Sleep stages and respiratory events were manually scored in accordance with the criteria of the American Academy of Sleep Medicine (AASM) Scoring Manual, Version 3 (2023).

Apnea was defined as a ≥90% reduction in airflow from baseline lasting at least 10 s, while hypopnea was defined as a ≥30% reduction in airflow lasting ≥10 s accompanied by ≥3% oxygen desaturation or an arousal. The apnea–hypopnea index (AHI) was calculated by dividing the total number of apnea and hypopnea events by the total sleep time, expressed as events per hour.

Participants were divided into four groups based on their AHI values:

- Normal (Control group): AHI < 5 events/h;
- Mild OSA: 5 ≤ AHI < 15 events/h;
- Moderate OSA: 15 ≤ AHI < 30 events/h;
- Severe OSA: AHI ≥ 30 events/h.

This PSG-based classification was used to evaluate the relationship between disease severity and the stiffness and thickness measurements of the genioglossus and geniohyoid muscles obtained using shear wave elastography (SWE).

### 2.3. Ultrasonography and Shear Wave Elastography Examination

The ultrasonographic and shear wave elastography examinations of the genioglossus and geniohyoid muscles were performed according to previously described protocols in the literature [12]. Patients were positioned supine with the head in slight extension. They were instructed to keep the tongue free in the mouth without touching the palate or retracting it.

To adequately visualize the target muscles, the transducer was first placed transversely in the midline of the submental region. Once the muscles were identified, the transducer was rotated to a longitudinal position, and thickness and elastography measurements of the right and left GG and GH muscles were performed sequentially. Care was taken to position the probe perpendicular to the skin without applying pressure.

Ultrasound examinations were performed on the same day before the polysomnography. Muscle thickness was measured using grayscale B-mode ultrasonography (US) as the anteroposterior distance between the superficial (anterior) and deep (posterior) fasciae of the muscle, after which shear wave elastography (SWE) measurements were conducted using the same high-frequency linear transducer, without changing the probe. Muscle elasticity was assessed at five different points using a 3 mm × 3 mm region of interest (ROI) placed away from fasciae and bony structures, and the results were recorded in meters per second (m/s). The mean value obtained from the five measurements was considered the stiffness value of the muscles.

**Figure 1:**
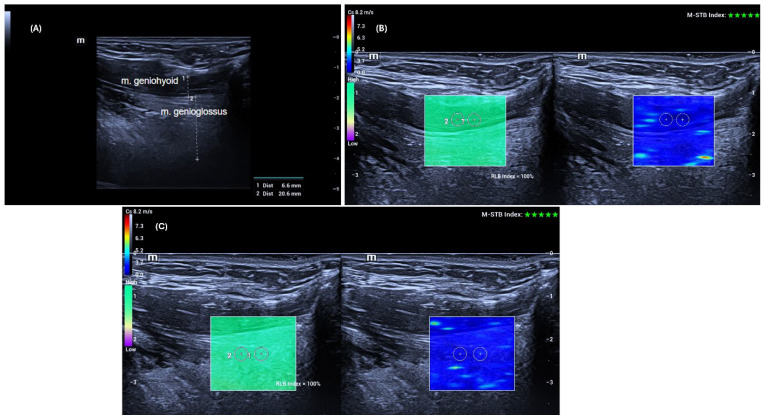
Geniohyoid and Genioglossus muscle thickness (**A**) was measured by the hypoechoic muscle tissue located between the superficial (anterior) and deep (posterior) fasciae of the muscle. Geniohyoid muscle elasticity (**B**) and Genioglossus muscle elasticity (**C**) measurements were taken in the regions of interest (ROI), with the ROI dimensions adjusted to 3 × 3 mm, and five measurements were obtained from each muscle. The ‘Motion Stability’ (M-STB) function was only measured at a 4–5-star level and in the presence of a green indicator. The ‘Reliability Map’ (RLB MAP) measurements were taken from areas with an RLB index of 90% or above.

All data were obtained using the SWE mode of the same US device (Mindray Resona I9, Shenzhen, China). Measurements were performed with a high-frequency linear transducer (L14-3 Ws) by a radiologist with 14 years of experience. Advanced technologies provided by the manufacturer supported the accuracy and stability of the measurements. The “Motion Stability” (M-STB) function allowed real-time monitoring of motion in the target area, and measurements were only recorded when a 4–5-star rating and green indicator were present. Additionally, image quality was assessed using the “Reliability Map” (RLB MAP) function, and measurements were taken only in areas with a reliability index of ≥90%. In cases of low signal quality or no signal, the measurement was considered invalid and repeated. Additionally, to increase measurement reliability, the interquartile range (IQR) and IQR/median ratios automatically calculated by the device were taken into account, and an IQR/median ratio of <0.30 was accepted as the standard [13]. Figure 1 shows an example of grayscale US and SWE measurements of the GG and GH muscles.

### 2.4. Statistical Analysis

Statistical analyses were performed using IBM SPSS Statistics (version 22.0; IBM Corp., Armonk, NY, USA). Continuous variables were expressed as mean ± standard deviation (SD). Normality was assessed using the Kolmogorov–Smirnov and Shapiro–Wilk tests, and homogeneity of variance was evaluated using Levene’s test. Comparisons between two groups were conducted using the independent samples *t*-test, while comparisons among four groups were performed using one-way analysis of variance (ANOVA). When a significant overall difference was detected, post hoc pairwise comparisons were performed using the Tukey HSD test to control for multiple comparisons. A *p*-value below 0.05 was considered statistically significant. Multivariate logistic regression analysis was performed to identify independent predictors of OSA presence, and ordinal logistic regression analysis was used to evaluate factors associated with disease severity.

## 3. Results

A total of 94 participants were included in the study (Group 0: *n* = 27; Group 1: *n* = 67). The mean age was similar between the groups (49.2 ± 12.5 vs. 52.9 ± 9.9 years, *p* = 0.223). There were no significant differences in sex distribution, height, weight, or BMI (all *p* > 0.05). However, the apnea–hypopnea index (AHI) was significantly higher in Group 1 compared to Group 0 (30.2 ± 22.3 vs. 2.6 ± 1.1, *p* < 0.001). The demographic and clinical characteristics of the participants are summarized in Table 1.

### 3.1. Morphometric Measurements

No significant differences were observed between the groups in terms of GG or GH muscle thickness on either the right or left side (all *p* > 0.05). Muscle thickness data are presented in Table 2. Similarly, subgroup analyses according to disease severity (mild, moderate, and severe OSA) revealed no statistically significant differences in muscle thickness (*p* > 0.05) (Table 3).

### 3.2. Elastography Measurements

Shear wave elastography (SWE) values showed significant differences between groups (Table 4). In m/s measurements, right GH elastography (2.7 ± 0.3 vs. 2.5 ± 0.3, *p* = 0.002), right GG elastography (2.7 ± 0.3 vs. 2.4 ± 0.2, *p* < 0.001), left GH elastography (2.7 ± 0.2 vs. 2.4 ± 0.2, *p* < 0.001), and left GG elastography (2.7 ± 0.3 vs. 2.4 ± 0.2, *p* < 0.001) were all significantly higher in Group 1 compared with Group 0. When stratified according to disease severity, right and left GH elastography values increased progressively from mild to severe OSA, with statistically significant differences (*p* < 0.05). No significant differences were observed in GG elastography across the severity subgroups (Table 5).

### 3.3. ROC Curve Analysis

**Figure 2:**
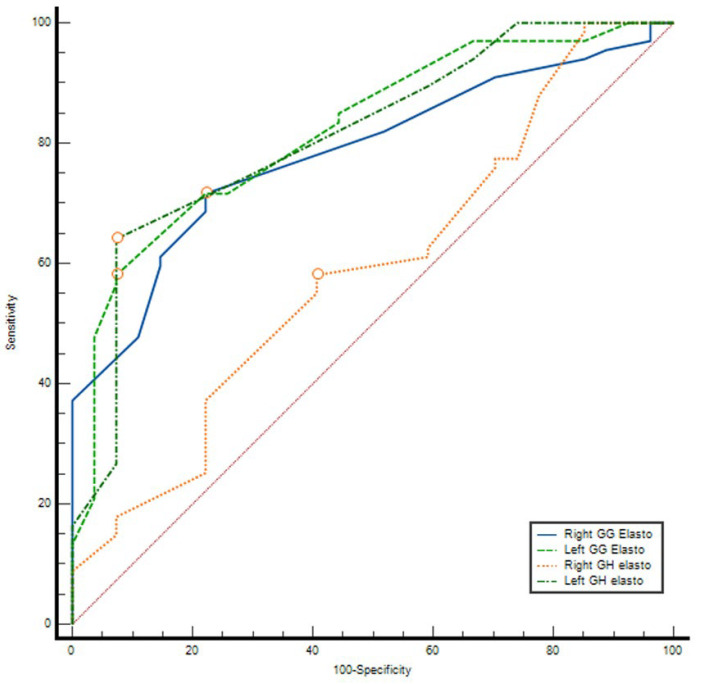
ROC curves demonstrating the diagnostic performance of geniohyoid (GH) and genioglossus (GG) muscle elastography measurements for distinguishing patients with obstructive sleep apnea from controls.

ROC analysis demonstrated that elastography parameters had variable diagnostic accuracy for distinguishing between groups. The AUC values were 0.591 (95% CI: 0.464–0.718, *p* = 0.169) for right GH elasto, 0.784 (95% CI: 0.691–0.877, *p* < 0.001) for right GG elasto, 0.813 (95% CI: 0.718–0.907, *p* < 0.001) for left GH elasto, and 0.821 (95% CI: 0.731–0.911, *p* < 0.001) for left GG elasto. The optimal cut-off values determined by the Youden index were 2.61 m/s, 2.525 m/s, 2.605 m/s, and 2.63 m/s, respectively. At these thresholds, sensitivity ranged between 58.2 and 71.6% and specificity between 59.3 and 92.6%. The highest discriminatory performances were observed for left GH elasto (AUC = 0.813) and left GG elasto (AUC = 0.821), with PPV values exceeding 95% and NPV ranging between 47 and 52% (Table 6) (Figure 2).

### 3.4. Correlation Analysis

Correlation analysis revealed significant positive associations between the AHI and all elastography parameters. AHI demonstrated moderate correlations with right GH elasto (r = 0.445, *p* < 0.001), right GG elasto (r = 0.378, *p* < 0.001), left GH elasto (r = 0.401, *p* < 0.001), and left GG elasto (r = 0.412, *p* < 0.001).

### 3.5. Regression Analysis

Multivariate logistic regression analysis showed that age was independently associated with the presence of OSA (β = 0.070, %95 CI: 0.003–0.136, *p* = 0.040). Male sex was also a significant predictor of OSA compared with female sex (β = 1.679, %95 CI: 0.306–3.052, *p* = 0.017). Among elastography parameters, left-sided GH elasto (β = 3.741, %95 CI: 0.396–7.087, *p* = 0.028) and left-sided GG elasto (β = 4.292, %95 CI: 0.681–7.903, *p* = 0.020) were identified as strong independent predictors of OSA presence. In contrast, BMI, right-sided GH elasto, and right-sided GG elasto were not significantly associated with OSA status (all *p* > 0.05). In the ordinal logistic regression analysis conducted among patients with OSA, right-sided GH elasto was significantly associated with increasing disease severity (β = 3.817, %95 CI: 1.325–6.309, *p* = 0.003). BMI also demonstrated an independent positive association with OSA severity (β = 0.149, %95 CI: 0.038–0.261, *p* = 0.009). Age showed a borderline association with disease severity (β = 0.053, %95 CI: −0.008–0.114, *p* = 0.086). No significant associations were observed for GG elasto parameters or sex in relation to OSA severity (all *p* > 0.05).

## 4. Discussion

In this study, the thickness of the genioglossus (GG) and geniohyoid (GH) muscles was evaluated using B-mode ultrasonography, and their elasticity properties were assessed using shear wave elastography (SWE) in adults diagnosed with OSA. While no differences were observed between groups in terms of muscle thickness, elasticity values were significantly higher in OSA patients compared with the non-OSA group. Although GG muscle elasticity did not show a significant increase with OSA severity, GH muscle elasticity—particularly on the right side—demonstrated a progressive increase corresponding to disease severity, and a significant difference was also observed on the left side between mild and severe OSA groups. These findings suggest that while thickness measurements of the extrinsic tongue muscles may not be informative for OSA diagnosis, elastography measurements that capture the biomechanical properties of these muscles could serve as a useful, noninvasive imaging modality for the assessment of OSA.

The lack of a statistically significant difference in GG and GH muscle thickness between OSA patients and the non-OSA group may suggest that pathological changes primarily occur at a microstructural level—such as alterations in collagen content, muscle fiber architecture, or fat infiltration—rather than in overall tissue thickness.

The significantly higher stiffness values of the GG and GH muscles in individuals with OSA compared to the non-OSA group suggest that these muscle tissues may become “stiffer.” This increased stiffness could elevate upper airway resistance, thereby predisposing to airway collapse. On the other hand, the lack of a significant relationship between GG stiffness and disease severity indicates that the elastic adaptation of GG tissue may not progress in parallel with OSA severity, or that other modulatory factors—such as neural control or muscle fatigue—may play a role. While GG elastography may not be sensitive for determining OSA severity, it could serve as a sensitive noninvasive marker for the diagnosis of OSA.

The correlation between increased stiffness of the right GH muscle and OSA severity, along with the significant difference observed between mild and severe groups on the left side, suggests that GH muscle stiffness may serve not only as a diagnostic marker for OSA but also as a biomechanical indicator sensitive to disease severity.

The different elasticity patterns observed between the geniohyoid and genioglossus muscles may reflect fundamental differences in the functional roles and mechanical loading characteristics of these muscles rather than the effects of chronic intermittent hypoxia alone. The geniohyoid muscle primarily plays a stabilizing role by maintaining the position of the hyoid bone and is therefore exposed to continuous and repetitive mechanical loading in OSA. When combined with intermittent hypoxia, this may promote progressive structural remodeling, including collagen accumulation and fibrosis, leading to a gradual increase in stiffness. In contrast, the genioglossus muscle is dynamically activated during respiration under strong central nervous control and provides effective neuromuscular compensation and load redistribution, which may limit excessive structural stiffening and lead to a plateau in stiffness despite increasing disease severity.

Most studies in the current literature have focused on tongue thickness and stiffness or have examined a single muscle (GG). In contrast, our study evaluates both the GG and GH muscles together, providing a novel perspective on their relationship with OSA. This approach allows for a more comprehensive assessment of the role of the extrinsic tongue muscles in the pathophysiology of OSA.

The GG muscle, the major dilator of the upper airway (UA), plays a critical role in the development of OSA [14]. When the GG contracts, multiple pharyngeal dilator muscles are synergistically activated, stabilizing and opening the UA. In chronic conditions such as OSA, the biomechanical properties and neural drive of tongue tissue may adapt in response to increased UA resistance and intermittent hypoxia. Additionally, an enlarged tongue volume can narrow the oral and pharyngeal cavities, which has been shown to be associated with OSA [15]. Therefore, evaluating the stiffness of the extrinsic tongue muscles through imaging may help monitor treatment effects and provide insights into the pathogenesis of OSA [7].

In the literature, there are no studies directly evaluating the extrinsic tongue muscles (particularly GG and GH) in OSA patients using ultrasonography or SWE. Therefore, it was not possible to directly compare our findings with studies on the same muscles. In a study by Liao et al., the overall thickness of the tongue muscle was reported to be increased in individuals with OSA, particularly in the severe OSA group [16]. In light of our finding of no significant differences in muscle thickness, this represents a contrasting observation in the literature.

Chang et al. reported significantly higher tongue stiffness in OSA patients compared with healthy controls in a study of 46 participants evaluated using US-SWE [7]. Similarly, in our study, GG and GH muscle stiffness was higher in OSA patients, with a significant increase in GH stiffness correlating with disease severity. Conversely, our findings contrast with those of Brown et al., who found lower tongue stiffness in OSA patients compared with controls using magnetic resonance elastography (MRE) [17]. The discrepancies between US and MRE measurements may be attributed to differences in the frequencies employed by each method. Additionally, Chu et al., using SWE, reported lower tongue stiffness in OSA patients and no correlation with disease severity [18]. These differences may arise from the fact that these studies focused on the overall tongue muscle rather than individual extrinsic muscles, such as GG and GH.

While most studies have examined the overall tongue tissue, our study evaluates both the genioglossus (GG) and geniohyoid (GH) muscles together, providing a novel perspective in the literature. This approach allows for a more comprehensive assessment of the role of the extrinsic tongue muscles in the pathophysiology of obstructive sleep apnea (OSA).

Suprahyoid muscle stiffness is differentially associated with OSA presence and severity. While increased left-sided GH and GG stiffness was independently related to disease presence, greater OSA severity was primarily associated with right GH stiffness and BMI. This lateralized and muscle-specific pattern may reflect asymmetric upper airway neuromuscular adaptation and highlights the potential role of targeted elastographic assessment of the suprahyoid muscles as an imaging biomarker in OSA.

One limitation of the study is the relatively small sample size within the subgroups, which may have limited the power to detect significant differences, particularly between severity groups. Another limitation is that, due to the small number of participants in the subgroups, analyses based on age were not performed. Considering these limitations, future studies are recommended to include larger and age-balanced samples. This would allow for a more comprehensive evaluation of the effects of OSA severity and age on the structural characteristics of the extrinsic tongue muscles.

## 5. Conclusions

In obstructive sleep apnea, upper airway collapse is the primary cause. The GG and GH muscles are responsible for maintaining airway patency, and ultrasonic elastography (SWE) provides an objective means to assess muscle stiffness and functional status. The elasticity properties of the GG and GH muscles may serve as a valuable noninvasive method for both diagnosing OSA and assessing its severity.

## Figures and Tables

**Table 1:** The  demographic data of the participants.

Variables	Group 0 (*n* = 27)	Group 1 (*n* = 67)	*p*
Age (years)	49.2 ± 12.5	52.9 ± 9.9	0.223
Sex (F/M)	10/17	23/44	0.601
Weight (cm)	171 ± 8	170 ± 9	0.834
Height (kg)	94.3 ± 17.9	94.6 ± 15.0	0.893
BMI	32.6 ± 6.2	32.7 ± 5.2	0.910
AHI	2.6 ± 1.1	30.2 ± 22.3	<0.001

BMI: Body Mass Index, AHI: Apnea–Hypopnea Index.

**Table 2:** Comparison of genioglossus (GG) and geniohyoid (GH) muscle thickness values between patients with and without obstructive sleep apnea (OSA).

Variables	Group 0 (Non-OSA) (*n* = 27)	Group 1 (OSA) (*n* = 67)	*p*
Right GH thickness (mm)	7.7 ± 1.8	8.2 ± 1.7	0.215
Right GG thickness (mm)	22.5 ± 3.2	22.5 ± 4.0	0.914
Left GH thickness (mm)	7.9 ± 1.8	8.2 ± 1.9	0.444
Left GG thickness (mm)	23.2 ± 3.8	22.9 ± 3.9	0.716

**Table 3:** Correlation of genioglossus (GG) and geniohyoid (GH) muscle thickness among groups classified by AHI in non-OSA and OSA patients.

Variables	Moderate (*n* = 22)	Severe (*n* = 26)	*p*
Right GH thickness	7.5 ± 1.2	8.7 ± 1.7	0.138
Right GG thickness	21.7 ± 2.3	22.9 ± 4.4	0.807
Left GH thickness	7.2 ± 1.6	8.9 ± 1.8	0.090
Left GG thickness	21.9 ± 2.7	23.2 ± 4.8	0.753

**Table 4:** Comparison of elasticity values of the genioglossus (GG) and geniohyoid (GH) muscles between patients with and without obstructive sleep apnea (OSA).

Variables	Group 0 (Non-OSA) (*n* = 27)	Group 1 (OSA) (*n* = 67)	*p*
Right GH elasticity value (m/s)	2.5 ± 0.3	2.7 ± 0.3	0.002
Right GG elasticity value (m/s)	2.4 ± 0.2	2.7 ± 0.3	<0.001
Left GH elasticity value (m/s)	2.4 ± 0.2	2.7 ± 0.2	<0.001
Left GG elasticity value (m/s)	2.4 ± 0.2	2.7 ± 0.3	<0.001

**Table 5:** Comparison of genioglossus and geniohyoid muscle elasticity values according to disease severity in patients with obstructive sleep apnea (OSA).

Parameter	Mild (*n*:19)	Moderate (*n*:22)	Severe (*n*:26)	*p*
Right GH elasticity value (m/s)	2.5 ± 0.2	2.6 ± 0.2	2.8 ± 0.2	<0.05 a,b,c
Right GG elasticity value (m/s)	2.6 ± 0.3	2.7 ± 0.3	2.8 ± 0.3	0.356
Left GH elasticity value (m/s)	2.6 ± 0.2	2.7 ± 0.3	2.8 ± 0.2	<0.05 b
Left GG elasticity value (m/s)	2.7 ± 0.2	2.7 ± 0.2	2.8 ± 0.3	0.421

a = *p* < 0.05 mild vs. moderate, b = *p* < 0.05 mild vs. severe, c = *p* < 0.05 moderate vs. severe.

**Table 6:** ROC Analysis Results.

Parameter	AUC (95% CI)	*p*	Cutoff Value	Sensitivity (%)	Specificity (%)	PPV (%)	NPV (%)
Right GH elasticity	0.591 (0.464–0.718)	0.169	2.61	58.2	59.3	78	36.4
Right GG elasticity	0.784 (0.691–0.877)	<0.001	2.525	71.6	77.8	88.9	52.5
Left GH elasticity	0.813 (0.718–0.907)	<0.001	2.605	64.2	92.6	95.6	51
Left GG elasticity	0.821 (0.731–0.911)	<0.001	2.63	58.2	92.6	95.1	47.2

## Data Availability

The data presented in this study are available from the corresponding author upon reasonable request. The data are not publicly available due to ethical and privacy restrictions.
